# *In Vitro* Drug Release and *Ex Vivo* Dermal Drug Permeation Studies of Selected Commercial Benzoyl Peroxide Topical Formulations: Correlation Between Human and Porcine Skin Models

**DOI:** 10.1021/acs.molpharmaceut.4c01058

**Published:** 2025-02-03

**Authors:** Murilo de Souza Brighenti, Lilian Rosário da Silva Montanheri, Marcelo Dutra Duque, Newton Andreo-Filho, Patricia Santos Lopes, Maria Teresa Junqueira Garcia, Lorraine Mackenzie, Vânia Rodrigues Leite-Silva

**Affiliations:** † Departamento de Ciências Farmacêuticas, Instituto de Ciências Ambientais, Químicas e Farmacêuticas, Universidade Federal de São Paulo, UNIFESP, Diadema 09913-030, Brazil; ‡ Clinical Health Sciences, University of South Australia, Adelaide, SA 5000, Australia; § Therapeutics Research Group, Frazer Institute, Faculty of Medicine, The University of Queensland, Brisbane QLD 4102, Australia

**Keywords:** skin penetration, benzoyl peroxide formulation, *in vitro* permeation test, *in vitro* release test, cutaneous permeation

## Abstract

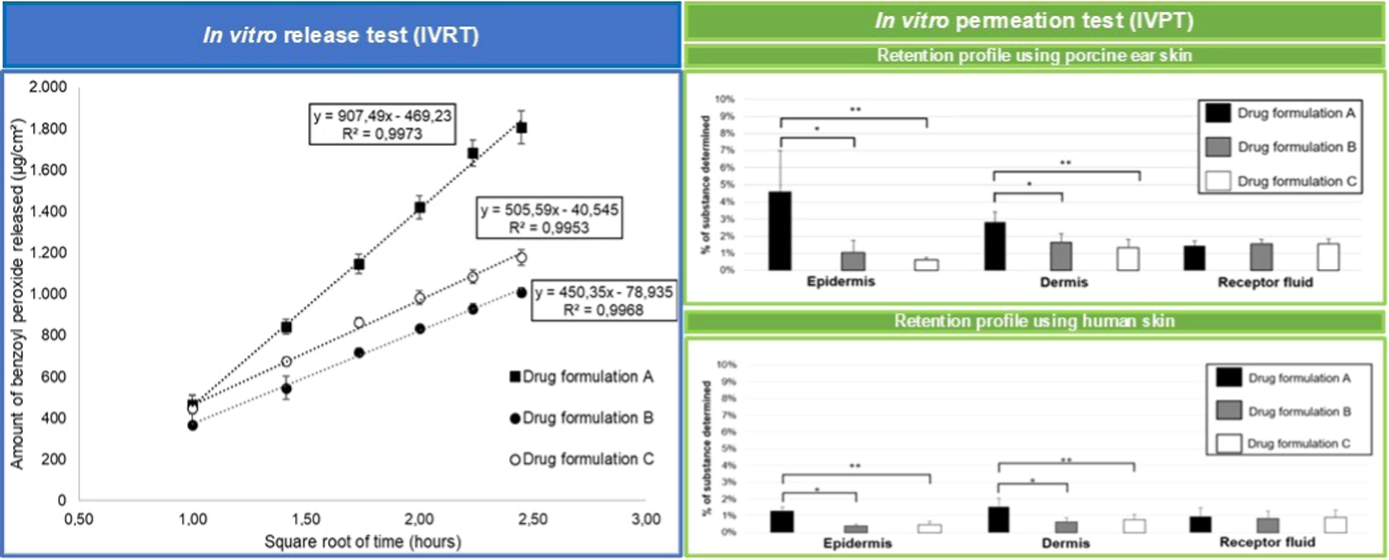

*In vitro* release testing (IVRT) serves as a crucial tool to assess the quality, physicochemical behavior, and performance of semisolid formulations already available on the market. *In vitro* skin permeation studies (IVPT) are widely used to evaluate the safety and efficacy profiles of topical drugs, utilizing biological membranes prepared from *ex vivo* human and porcine skin tissues. This study aimed to develop and validate a discriminative IVRT method to evaluate various marketed topical benzoyl peroxide formulations. Additionally, IVPT was employed to assess skin permeation and retention profiles of these formulations, comparing porcine skin results with those obtained by using *ex vivo* human skin tissues. Physicochemical differences among the evaluated benzoyl peroxide formulations were identified, with the poloxamer-based formulation exhibiting a higher release rate. IVPT using both porcine and human skin differentiated retention and skin permeation profiles, with the poloxamer-based formulation demonstrating greater skin retention capacity compared to the other formulations evaluated. Similar conclusions on benzoyl peroxide retention and cutaneous permeation were drawn from both porcine and human skin IVPT tests, confirming the correlation between the two models.

## 1 Introduction

Acne vulgaris is a chronic inflammatory skin condition characterized by inflammation of the pilosebaceous glands, developing into open comedones (blackheads), closed comedones (whiteheads), and inflammatory lesions such as nodules, pustules, and papules.^1^ Certain species of bacteria, *Cutibacterium* and *Staphylococcus*, are associated with this condition. In addition to the discomfort, emotional stress, disfigurement, and formation of permanent scars on the skin that can result from the development of acne, disorders and psychological problems such as low self-esteem, depression, and anxiety can directly and negatively interfere with the patient’s quality of life.^2^

One of the most commonly prescribed topical treatments for atopic acne vulgaris is benzoyl peroxide. When applied to the skin surface, the lipophilic benzoyl peroxide quickly permeates through the outermost layer, the stratum corneum, and/or the follicular openings. Within the skin layers it is then metabolized to benzoic acid that is absorbed systemically and then excreted in the urine after being absorbed into the systemic circulation.^3−5^ In the follicle, prior to its metabolism, benzoyl peroxide acts as a potent generator of free radicals.^5^ These moieties are capable of oxidizing bacterial proteins present in the epidermis, and thus considerably reducing the bacterial load, in addition to having keratolytic and anti-inflammatory activities.^6,7^ While the majority of benzoyl peroxide, topically applied for the treatment of acne, reportedly remains on the surface of the skin, with only 5 to 10% absorbed systemically in the form of benzoic acid following a 24-h exposure,^6^ how the active ingredient is formulated may significantly affect this absorption. It is necessary to understand and evaluate both the cutaneous permeation and systemic absorption of these formulations to evaluate their relative safety.

*In vitro* skin permeation studies (IVPT) are widely used to assess the safety and efficacy profiles of topical drugs using biological membranes prepared from *ex vivo* human skin (biological waste from abdominoplasty surgeries).^8^ The procurement of human skin is often not possible or is time-consuming and laborious, involving ethical consents and reporting, although it is the “gold standard” for these measurements. The use of animal membranes *e.g.*, porcine ear skin, is deemed an acceptable substitute due to its physiological and histological similarities to human skin including importantly the density of hair follicles.^8−10^

The novelty of this work lies in the development and validation of a discriminative method to evaluate the *in vitro* drug release profiles of different 5% benzoyl peroxide topical formulations. Moreover, skin permeation and retention profiles using IVPT of the same benzoyl peroxide-containing formulations were assessed by comparing porcine skin results with those obtained using *ex vivo* human skin.

## 2 Materials and Methods

Four topical formulations currently on the market from different manufacturers were obtained over the counter for these studies. Three of the products (designated A–C) each contained 5% benzoyl peroxide with the fourth (D), identical to formulation A except that it contained 10% benzoyl peroxide. Formulations with their excipients are given in Table 1.

**Table 1:** Qualitative Composition of Each Formulation Tested^a^

drug formulation A (5% BP)	drug formulation B (5% BP)	drug formulation C (5% BP)	drug formulation D (10% BP)
poloxamer	stearic acid	stearic acid	poloxamer
silicon dioxide	mineral oil	cetyl alcohol	silicon dioxide
sodium docusate	anionic self-emulsifying wax	dimethyl isosorbide	sodium docusate
EDTA	methylparaben	simethicone	EDTA
propylene glycol	propylene glycol	parfum	propylene glycol
Carbopol 940	sodium citrate	cetomacrogol 1000	carbopol 940
sodium hydroxide	xanthan gum	benzoyl peroxide	sodium hydroxide
glycerol	veegum regular	purified water	glycerol
acrylate copolymer	benzoyl peroxide		acrylate copolymer
benzoyl peroxide	purified water		benzoyl peroxide
purified water			purified water

a BP = benzoyl peroxide.

All solvents and reagents used for experiments were of high-performance liquid chromatography (HPLC) grade. Methanol, acetonitrile, acetic acid, potassium phosphate monobasic anhydrous, sodium dibasic phosphate anhydrous, and sodium chloride were purchased from Merck (São Paulo-SP, Brazil) and tetrahydrofuran was purchased from Sigma-Aldrich (São Paulo-SP, Brazil). A characterized standard (Campinas-SP, Brazil) and USP reference standard (São Paulo-SP, Brazil) were used for the quantification of benzoyl peroxide and benzoic acid, respectively. Ultrapure water was obtained from a Milli-Q purification system (Millipore, Advantage A10, Brazil).

### 2.1 *In Vitro* Release Test (IVRT)

An IVRT method to determine and compare benzoyl peroxide release rates from the different formulations was developed, validated for linearity, precision, reproducibility, selectivity, sensitivity, recovery, and *sink* conditions, and performed in accordance with United States Pharmacopeia 2024 General Chapter ⟨1724⟩.^11^ Franz vertical diffusion cells (receptor volume of 21 mL) and a Phoenix RDS automatic sampling collection system were used (Teledyne Hanson, São Paulo, Brazil). Nylon was used as a synthetic membrane (diameter 25 mm, pore size 0.45 μm, Merck, Brazil) and a degassed mixture of acetonitrile:water (50:50, v/v) was used as a receptor medium, as the solubility of benzoyl peroxide was 2.0 mg/mL in this receptor fluid, which is >10 times the maximum expected concentration, this ensured sink conditions.^12,13^ The use of this receptor fluid is justified because benzoyl peroxide is poorly soluble in water, (0.0091 mg/mL at 25 °C^14^) and is not compatible with acetone, methanol, or ethanol.^15^

Synthetic membranes were equilibrated in the receptor medium for 30 min before being mounted in Franz cells. After assembly and filling with the receptor medium, the system was equilibrated for an additional 30 min with agitation at 600 rpm. A pseudoinfinite dose, about 700 mg of each formulation A–D (*N* = 6 per formulation), was applied in the donor chamber of each Franz cell (diffusional area 1.77 cm^2^). At time points: 1, 2, 3, 4, 5, and 6 h, 500 μL of the receptor fluid was removed for testing and replaced immediately with the fresh warmed receptor medium. Temperature was maintained at 32 ± 1 °C throughout the test. Collected samples were stored at room temperature prior to being analyzed by a developed and validated HPLC method to determine the concentration of benzoyl peroxide in the receptor fluids.

### 2.2 *In Vitro* Permeation Test (IVPT)

IVPT was conducted using two biological membranes, human skin obtained from abdominoplastic surgery and unscalded porcine ear skin according to OECD 428 guidance.^16^*Ex vivo* human skin and porcine ear skin were obtained after approval by the appropriate Ethics Committees of UNIFESP (Protocol number: 5.907.064 and 2581110422, respectively). Porcine ear skin was supplied by a reliable slaughterhouse (Capivari, São Paulo, Brazil).

The adipose tissue was removed, leaving full-thickness membranes for both types of skin. Skin integrity was verified by the measurement of transepidermal water loss (TEWL; Tewameter TM 300 Courage + Khazaka Electronic) with values below 10.0 and 35.0 g/m^2^/h for human and porcine skin, respectively, considered as satisfactory.^15^ Franz vertical diffusion cells (4.5 mL) were mounted with the skin membranes between the donor and receptor chambers (stratum corneum facing the donor chamber), *N* = 5 per skin type per formulation. The receptor chambers were filled with phosphate-buffered saline (PBS) pH 7.4, used as the receptor fluid due to the high solubility of benzoic acid in aqueous solutions and to ensure sink conditions the solubility of benzoic acid was 4.25 mg/mL in this receptor fluid, which is >3.8 times the maximum expected permeation concentration.^12^ Franz cells were then equilibrated at 32 ± 1 °C for 30 min after which formulation A, B, or C was applied (100 mg, finite dose) in the donor compartment (diffusional area of 1.3 cm^2^) of each Franz cell, resulting in a dose of 3.85 mg of benzoyl peroxide/cm^2^. The donor chambers were left open to provide nonocclusive conditions and temperature was maintained at 32 ± 1 °C throughout the test. Samples of the receptor fluid (500 μL) were removed at set time points over 24 h and replaced with the fresh, prewarmed (32 ± 1 °C) receptor fluid.

After the 24-h sample was taken, the cells were dismantled and the surface of the skin was wiped with cotton buds to remove any remaining formulation. To separate the epidermis and dermis, the skin was transferred to a small plastic bag and heated in a water bath at 60 °C for 90 s. Tweezers were then used to mechanically separate the epidermis from the dermis. Epidermis was extracted with an acetonitrile:water (80:20, v/v) solution. Dermis was chopped into small pieces and extracted with an acetonitrile:water (80:20, v/v) solution. The cotton buds were extracted with 10 mL of a tetrahydrofuran:acetonitrile:water (70:20:10, v/v/v) solution. All samples were shaken on a vortex shaker and sonicated for 30 min prior to being analyzed by HPLC.

### 2.3 Analytical Methods

The HPLC System used to develop and validate the methods and analyze the *in vitro* drug permeation and *in vitro* drug release profiles of the samples was equipped with a separation module Agilent 1260 that consisted of a quaternary pump (G1311C), an autosampler (G1329B), and a photodiode array detector (G4212B).

Benzoyl peroxide and benzoic acid concentrations generated during IVRT and IVPT studies were measured using ultraviolet (UV) detection at 244 nm and an XBridge C18 column (Waters, 5 μm, 250 mm × 4.6 mm) maintained at 35 °C. For IVRT samples the mobile phase consisted of a methanol:water mixture (76:24, v/v, with pH adjusted to 3.0 using acetic acid) in the isocratic mode, with an injection volume of 2 μL and a flow rate of 1.2 mL/min. The run time was 10 min and the approximate retention time for benzoyl peroxide was 6.2 min. For IVPT receptor fluid samples, a gradient elution mode was used. Mobile phase A (MP A) consisted of a water:methanol mixture (80:20, v/v, with pH adjusted to 3.0 using acetic acid), and mobile phase B (MP B) consisted of HPLC grade methanol. The elution started with a mobile phase ratio of 65:35 v/v (MP A:MP B), it was isocratic for the first 8 min and was altered gradually to 5:95, v/v (MP A:MP B) over 12 min. This composition was maintained for an additional 10 min, after which the initial eluent composition was restored in 10 min. The injection volume was 10 μL with a flow rate of 1.2 mL/min. Run time was 40 min, and approximate retention times for benzoic acid and benzoyl peroxide were 5.9 and 19.1 min, respectively.

To measure the concentration of benzoic acid and/or benzoyl peroxide in the different matrices of the experimental samples *i.e.*, unabsorbed product from the donor chamber and skin surface, extractions from different skin layers, and the receptor medium, analytical curves were constructed over the following ranges: 0.25 to 0.75 mg/mL and 1 to 50 μg/mL for benzoyl peroxide and 0.2 to 30.0 μg/mL for benzoic acid. The chromatographic method used to quantify the samples from the IVRT study was validated in accordance with the ICH Q2 guideline.^17^

### 2.4 Statistical Analysis

The results were reported as the mean ± standard deviation (SD). The release results were analyzed using Mann–Whitney U nonparametric statistical test, with a 90% confidence interval.^11^ The permeation results were analyzed using one-way ANOVA followed by Tukey’s multiple comparison test. Values were considered significantly different when *p* < 0.05. In both analyses, Minitab software, version 18.1, was used.

## 3 Results and Discussion

### 3.1 IVRT Results

**Figure 1:**
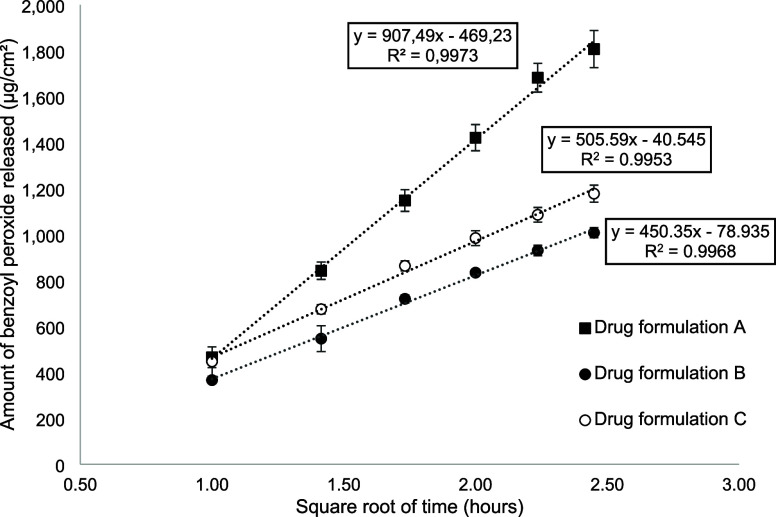
Average benzoyl peroxide release profiles for drug formulations A–C, each containing 5% benzoyl peroxide. Data represent mean ± SD of 6 replicates, and the linear regression of mean.

The concentrations of benzoyl peroxide measured in each of the receptor fluid samples were used to calculate the amount of benzoyl peroxide released from each formulation (μg/cm^2^). When this amount released is plotted against the square root of time in hours (Higuchi model) the slope of the linear regression, represents the release rate of benzoyl peroxide from each of the formulations^18,19^ (Figure 1). The benzoyl peroxide release rate was demonstrated to be significantly higher for topical formulation A than for formulations B and C, which were not statistically different from each other (Mann–Whitney U nonparametric statistical test, Table 2). Formulations that present results within the acceptable range of 75–133.33% are considered similar.^11^

**Table 2:** Equivalence of the Tested Formulations—Mann–Whitney U Test^11^

	CI 90%		
pairwise comparison	lower limit (%)	upper limit (%)	sameness confirmed?
drug formulation A *vs* drug formulation B	45	49.00	no
drug formulation A *vs* drug formulation C	50	57.06	no
drug formulation B *vs* drug formulation C	106	122.84	yes

As shown in Figure 1, the *in vitro* release profiles demonstrate that the semisolid formulation A (poloxamer-based formulation) exhibited a higher release rate of benzoyl peroxide compared to formulations B and C (oil-based matrices). This higher release rate is likely due to the physicochemical properties of benzoyl peroxide (log *P* = 3.5), significantly contributing to a lower chemical affinity between the drug and the hydrophilic matrix.^20^ A study conducted by Özer et al. demonstrated similarly that gel-based formulations containing benzoyl peroxide showed higher *in vitro* release rates compared to different oil-based matrices.^21^

### 3.2 IVPT Results

IVPT experiments were conducted to identify potential permeation differences between three marketed topical formulations, each containing 5% benzoyl peroxide (Table 1). Experiments were conducted using two distinct biological membrane models: *ex vivo* full-thickness human skin and porcine skin. The amount of nonpermeated benzoyl peroxide (remaining in the donor chamber at the end of the experiments), the amount of benzoyl peroxide retained in the skin layers (epidermis and dermis), and the amount of benzoic acid (marker) that permeated the membranes throughout the 24 h of the study.^3,4^

**Figure 2:**
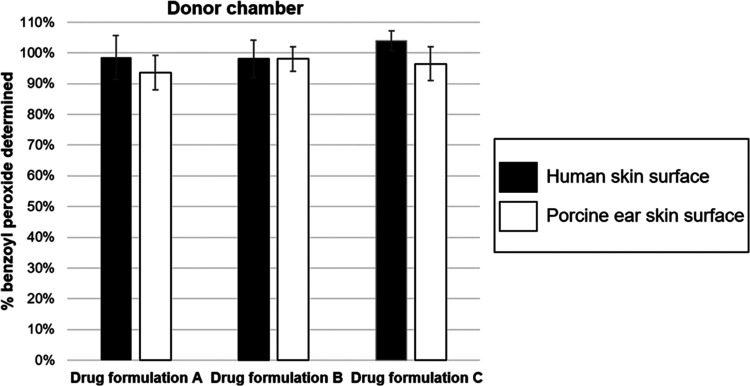
Benzoyl peroxide remaining on the skin surface at completion of the IVPT experiments, expressed as a percentage (mean ± SD) of the applied dose to *ex vivo* human and porcine skin.

Figure 2 shows the percentage (mean ± SD) of benzoyl peroxide measured in samples from the donor chamber, *i.e.*, remaining on the surface of porcine and human skin at the end of the IVPT experiments, relative to the applied dose.

The unabsorbed dose, expressed as a percentage of the applied dose, of benzoyl peroxide from formulations A, B, and C (measured in the donor compartment or remaining on the skin surface) following 24 h show that the majority (>92%) of benzoyl peroxide was retained on the surface of the skin, regardless of the biological membrane used (Figure 2). This work is consistent with that of Nacht et al., who reported that following *ex vivo* experiments using excised human skin as the biological membrane, 95.5% of the topically applied dose of formulations containing 10% benzoyl peroxide remained on the skin surface at the end of the 8-h test period.^3^ For the IVPT mass balance was achieved for each evaluated topical drug formulation meeting the acceptance criteria (90.0–110.0%) established in OECD Test Guideline 428^16^ for both *ex vivo* human and porcine skin. For human skin, the mass balance obtained (mean ± SD) for topical drug formulations A, B, and C were 102.4 ± 7.4, 100.0 ± 5.9, and 106.1 ± 3.7%, respectively, and for porcine skin, 102.4 ± 3.7, 103.1 ± 3.4, and 100.0 ± 5.0%, respectively.

#### 3.2.1 Permeation and Retention Profile Using the Porcine Skin Model

**Figure 3:**
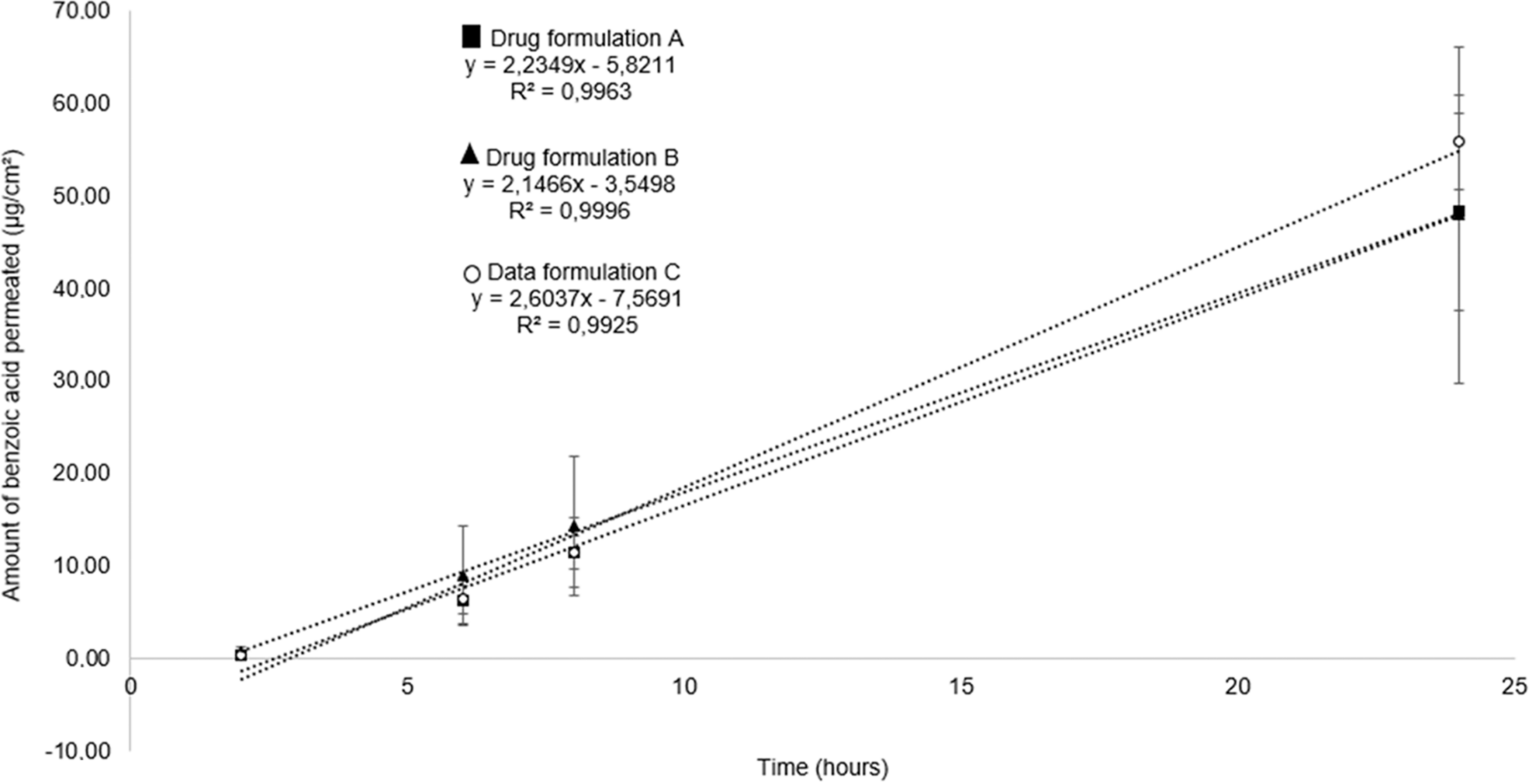
Permeation profiles through *ex vivo* porcine skin of different marketed formulations containing 5% benzoyl peroxide, measuring the marker benzoic acid collected in the receptor fluid over time. Data represent mean ± SD of 5 replicates and the linear regression of the mean.

The permeation profiles through *ex vivo* porcine skin of different marketed formulations containing 5% benzoyl peroxide are shown in Figure 3.

The permeation parameters of flux, total amount of drug permeated over 24 h, and lag time (time for benzoic acid to be measurable in the receptor fluid), obtained from IVPT experiments using *ex vivo* porcine skin are presented in Table 3. Statistical analysis of the data (Figure 3 and Table 3) shows no significant differences between any of the permeation parameters measured for the three drug formulations containing 5% benzoyl peroxide tested.

**Table 3:** Permeation Parameters for Benzoyl Peroxide-Containing Drug Formulations Generated by IVPT Experiments Using *Ex Vivo* Porcine Skin

drug formulation	*J*^a^ (μg/cm^2^/h)	*A*_total_^b^ (μg)	*T*_lag_ (h)^c^
A	2.23 ± 0.48	87.93 ± 21.76	2.68 ± 0.63
B	2.15 ± 0.78	95.32 ± 41.09	1.88 ± 1.14
C	2.60 ± 0.25	98.25 ± 8.71	2.89 ± 0.47

a *J* = permeation flux.

b *A*_total_ = total amount permeated;

c *T*_lag_ = lag time (h); values are mean ± SD, *N* = 5.

**Figure 4:**
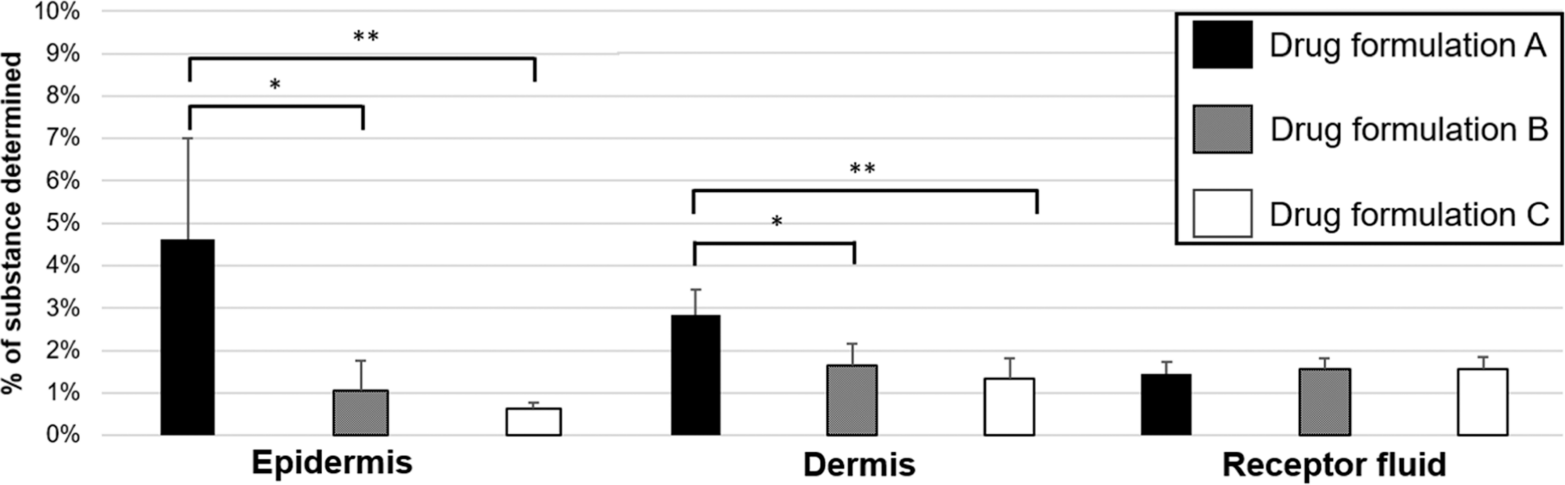
Amount of benzoyl peroxide measured in the epidermis and the dermis or benzoic acid in the receptor fluid expressed as a percentage of the applied dose to *ex vivo* porcine skin. Data represent mean ± SD of 5 replicates. *Indicates statistical significance (*p* < 0.05).

Figure 4 shows the concentration of benzoyl peroxide retained in the *ex vivo* porcine skin layers (epidermis and dermis) and the metabolite benzoic acid that permeated to the receptor fluid expressed as a percentage of the applied dose for each of the three drug formulations.

The results presented in Figure 4 show that less than 2% of the benzoyl peroxide (measured as the metabolite benzoic acid) permeated the receptor fluid. Additionally, it was not possible to detect any benzoyl peroxide in the receptor fluid demonstrating that the benzoyl peroxide was completely metabolized before completing its passage through the skin (detection limit of 7.5 ppb).

The benzoyl peroxide in drug formulation A had greater cutaneous retention compared to the other evaluated semisolid formulations for both the epidermal and dermal skin layers (Figure 4 and Table 4). This could be attributed to the increased release rate from this formulation providing a higher concentration of benzoyl peroxide available to interact and diffuse through the stratum corneum, as lipophilic molecules demonstrate greater ease in diffusing and penetrating this layer.^22^

**Table 4:** Amount of Benzoyl Peroxide Retained in Porcine Skin Following IVPT^a^

skin layers	drug formulation A	drug formulation B	drug formulation C
epidermis	199.36 ± 85.11 μg^b^	35.55 ± 17.32 μg	32.02 ± 10.18 μg
dermis	63.56 ± 11.53 μg^b^	39.37 ± 11.09 μg	32.56 ± 13.33 μg

a Data presented as mean ± SD, (*N* = 5).

b Demonstrates *p* < 0.05 compared to the other drug formulations.

#### 3.2.2 Permeation and Retention Profile Using the Human Skin Model

**Figure 5:**
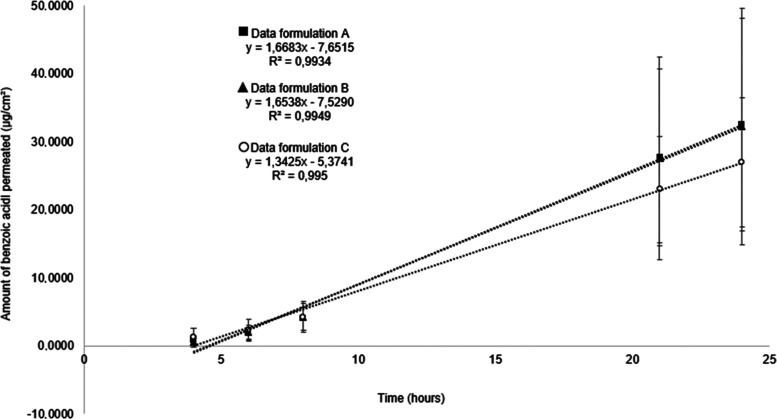
Permeation profiles through *ex vivo* human skin of different marketed formulations containing 5% benzoyl peroxide, measuring the marker benzoic acid collected in the receptor fluid over time. Data represent mean ± SD of 5 replicates, and the linear regression of the mean.

The permeation profiles through *ex vivo* human skin of different marketed formulations containing 5% benzoyl peroxide are shown in Figure 5.

The permeation parameters of flux, total amount of drug permeated over 24 h, and lag time obtained from IVPT experiments using *ex vivo* human skin are presented in Table 5. Statistical analysis of the data (Figure 5 and Table 5) shows no significant differences between any of the permeation parameters measured for the three semisolid drug formulations containing 5% benzoyl peroxide tested. These findings were the same as those for IVPT using *ex vivo* porcine skin presented in Figure 3 and Table 3. Zeichner et al. demonstrated that, on comparing different formulations containing 2.5 and 5% benzoyl peroxide through *ex vivo* permeation assays using human skin, the total amount of benzoic acid reaching the receptor medium was the same after 24 h.^23^ Similar results were obtained by Özer et al., who conducted *ex vivo* assays using excised rat skin as the biological membrane, showing comparable permeation flux of benzoyl peroxide for cream and gel formulations.^21^

**Table 5:** Permeation Parameters for Benzoyl Peroxide-Containing Drug Formulations Generated by IVPT Experiments Using *Ex Vivo* Human Skin

drug formulation	*J*^a^ (μg/cm^2^/h)	*A*_total_^b^ (μg)	*T*_lag_ (h)^c^
A	1.52 ± 0.80	83.30 ± 41.19	4.32 ± 0.50
B	1.60 ± 0.90	86.06 ± 47.49	4.52 ± 0.20
C	1.32 ± 0.43	79.08 ± 27.33	3.74 ± 0.65

a *J* = permeation flux.

b *A*_total_ = total amount permeated;

c *T*_lag_ = lag time (h); values are mean ± SD, *N* = 5.

Permeation parameters including flux, total amount permeated, and lag time obtained from IVPT experiments using *ex vivo* human skin are presented in Table 5.

**Figure 6:**
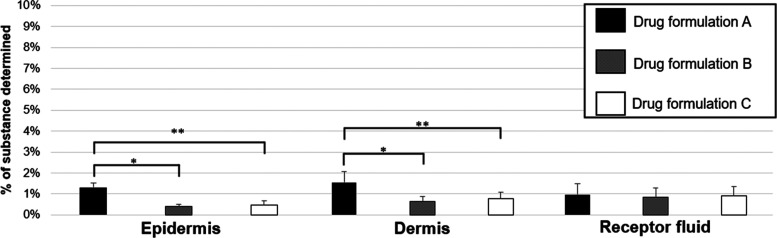
Amount of benzoyl peroxide measured in the epidermis and the dermis or benzoic acid in the receptor fluid expressed as a percentage of the applied dose to *ex vivo* porcine skin. Data represent mean ± SD of 5 replicates. *Indicates statistical significance (*p* < 0.05).

Figure 6 shows the concentration of benzoyl peroxide retained in the *ex vivo* human skin layers (epidermis and dermis) and the metabolite benzoic acid that permeated to the receptor fluid expressed as a percentage of the applied dose for each of the three drug formulations

The results presented in Figure 6 show that less than around 1% of the benzoyl peroxide (measured as the metabolite, benzoic acid) permeated the receptor fluid. Additionally, it was not possible to detect any benzoyl peroxide in the receptor fluid demonstrating that benzoyl peroxide was completely metabolized before completing its passage through the human skin. This is in agreement with the findings from the porcine skin IVPT experiments reported here, although slightly less benzoic acid was measured in the receptor fluid. These results are also in agreement with the report by Nacht et al. who, following administration of radiolabeled ^14^C-benzoyl peroxide to *ex vivo* human skin recovered only benzoic acid from the dermal side of the skin and when topically applied to rhesus monkey *in vivo* they failed to detect hippuric acid in the urine, that would have been indicative of hepatic metabolism, and favored hepatic clearance.^3^ Again, with the use of radiolabeled ^14^C-benzoyl peroxide, but formulated as a 10% gel and applied to hairless rat skin, Wepierre et al. described the conversion to and elimination of benzoic acid over the same time frame reported here.^24^Table 6 presents the amount of benzoyl peroxide retained in the different skin layers.

**Table 6:** Amount of Benzoyl Peroxide Retained in Human Skin Following IVPT^a^

skin layers	drug formulation A	drug formulation B	drug formulation C
epidermis	55.36 ± 14.35 μg^b^	18.96 ± 5.34 μg	18.37 ± 7.36 μg
dermis	32.44 ± 10.98 μg^b^	15.26 ± 5.67 μg	15.56 ± 4.22 μg

a Data presented as mean ± SD, (*N* = 5).

b Demonstrates *p* < 0.05 compared to the other drug formulations.

The benzoyl peroxide in drug formulation A had greater cutaneous retention compared to the other evaluated semisolid formulations for both the epidermal and dermal skin layers (Figure 6 and Table 6), results similar to and supporting those reported for the porcine skin.

Topical drug formulation A presents physicochemical properties that probably allow a greater release of benzoyl peroxide, as shown in the IVRT experiments, and thus provides greater availability of benzoyl peroxide to interact with the stratum corneum. This in turn would favor a greater amount of benzoyl peroxide deposition in the epidermis and dermis layers, without underestimating the absorption-promoting effect that some component of the formulation can exert. Alternately, topical drug formulations B and C, showed a lower release flow of benzoyl peroxide, resulting in a lower deposition.^18−20,25^ Benzoyl peroxide is known to display great bactericidal efficacy against the *C.acnes* pathogen and with a greater amount of drug formulation A retained in the hair follicles, it is expected that this would result in greater effectiveness for the topical treatment of acne by this formulation.^23,25^

The results from these topical application studies, specifically the retention of benzoyl peroxide in the skin and its metabolism within the skin to benzoic acid eliminated by renal clearance once it reaches the systemic blood circulation in the dermis, suggest that the behavior of drug formulations A, B, and C were similar, even when using two different biological membranes, fresh *ex vivo* pig skin and fresh *ex vivo* human skin. It is known that porcine skin is the animal model that most resembles human skin histologically, with similar thickness or the main barrier layers of the skin: stratum corneum and epidermis.^26^ However, it is important to note that pig skin did allow greater permeation of benzoyl peroxide compared to human skin both here and in other studies.^27^

## 4 Conclusions

The developed IVRT method presented linearity, precision, reproducibility, accuracy, selectivity, sensitivity, recovery, and suitable sink conditions. This method was able to differentiate between three formulations of marketed topical medications containing 5% benzoyl peroxide, presumably due to their physicochemical differences; therefore, it can be concluded that the method presented adequate discriminatory power.

By conducting IVPT tests using porcine skin and human skin as biological membranes, it was possible to evaluate and identify differences in the retention and skin permeation profile between the three marketed formulations tested.

The IVPT tests demonstrated equivalence for all of the evaluated benzoyl peroxide-containing marketed drug formulations regarding their cutaneous permeation and skin retention capacity using both *ex vivo* porcine and human skin preparations. Thus, porcine skin models offer an alternative for studying the *ex vivo* permeation profiles of topical drug products.

## Supporting Information Available

Analytical method validation (HPLC and *in vitro* release); benzoyl peroxide solubility; compatibility evaluation of synthetic membrane and benzoyl peroxide; linearity; precision and reproducibility; sensitivity; and selectivity and recovery (PDF)
