# Supplementary material for: In Vitro Drug Release and Ex Vivo Dermal Drug Permeation Studies of Selected Commercial Benzoyl Peroxide Topical Formulations: Correlation Between Human and Porcine Skin Models
Source: Mol Pharm. 2025 Feb 3;22(3):1365–72. doi: 10.1021/acs.molpharmaceut.4c01058 (PMC11881039; doi:10.1021/acs.molpharmaceut.4c01058)
Supplement: Supplementary file 1 — mp4c01058_si_001.pdf [file mp4c01058_si_001.pdf]

Supporting Information

***In vitro* Drug Release and *ex vivo* Dermal Drug Permeation Studies of Selected Commercial Benzoyl Peroxide Topical Formulations: Correlation Between Human and Porcine Skin Models**

Murilo de Souza Brighenti[a], Lilian Rosário da Silva Montanheri[a], Marcelo Dutra Duque[a], Newton Andreo-Filho[a], Patricia Santos Lopes[a], Maria Teresa Junqueira Garcia[a], Lorraine Mackenzie[b], Vânia Rodrigues Leite-Silva[a,c]*

[a] Departamento de Ciências Farmacêuticas, Instituto de Ciências Ambientais, Químicas e Farmacêuticas, Universidade Federal de São Paulo, UNIFESP-Diadema, Brazil;
[b] Clinical Health Sciences, University of South Australia, Adelaide, SA 5000, Australia;
[c] Therapeutics Research Group, Frazer Institute, Faculty of Medicine, The University of Queensland, Brisbane, QLD 4102, Australia;

*Corresponding author: vania.leite@unifesp.br.

Analytical method validation – *In vitro* release test

The results obtained for the validation of the chromatographic method are summarized in the **Table SI1.**

Table SI1. Results from the validation of the chromatographic method used to analyze IVRT samples.

**Concentration range:**	14, 36, 72, 110, 145, 180 and 220 μg/mL
**Linearity**	$R^2 \geq 0,99$
**Precision**	CV ≤ 15%
**Accuracy**	Recoveries obtained within the range of 90 to 110%
**LOQ, LOD:**	14 and 0.07 μg/mL, respectively
**Stability:**	36 hours at 32 °C
**Specificity:**	Peak purity ≥ 990

The validation parameters for the HPLC method complied with the pre-defined acceptance criteria in accordance with the ICH Q2 guideline[1].

Benzoyl peroxide solubility in receptor medium

The choice of acetonitrile:water (50:50, v/v) receptor medium was made after literature review. Benzoyl peroxide is a hydrophobic compound; therefore, it was decided to use 50% of acetonitrile in the composition to guarantee adequate sink conditions for IVRT [2,3].

The solubility of the benzoyl peroxide in the receptor medium adopted for conducting the *in vitro* release assays was evaluated in triplicate. Glass vials with lids suitable for conducting the assay were used. In each glass vial, a sufficient amount of the drug (approximately 2 g of the drug in 100 mL of receptor medium) was added to obtain a saturated solution, where the presence of precipitate could be observed with the naked eye. The three saturated solutions were incubated in an orbital shaking incubator "shake-flask" (Model MA832, Marconi®), maintained at 32 ± 1 °C for 6 hours at 150 rpm [2,3]. After the total time of 6 hours, aliquots of the supernatant from each solution were withdrawn and filtered (Nylon syringe filter, 0.45 µm, 25 mm, Merck-Millipore®) and injected into the chromatographic method described in item 2.3. The results obtained are presented in **Table SI2**.

Table SI2. Solubility results for benzoyl peroxide in the receptor medium adopted after 6 hours of evaluation.

Solution	Concentration obtained in mg/mL
Sample 1	1.9167
Sample 2	2.0160
Sample 3	2.0606
Average	1.9978
SD	0.0737
RSD (%)	3.7

The average obtained for the sixth collection was 0.1523 mg/mL of benzoyl peroxide, therefore, knowing that the solubility of the drug in question in the adopted receptor medium is 1.9978 mg/mL, it is possible to conclude that the receiving medium adopted acetonitrile:water (50:50, v/v) presents a sink condition of approximately 13 times, presenting satisfactory solubility for conducting *in vitro* release tests [2,3,4].

Compatibility evaluation of synthetic membrane and benzoyl peroxide stability in receptor medium

In order to evaluate the possible interaction of benzoyl peroxide, the active pharmaceutical ingredient (API) in the formulations to be tested, with the synthetic membrane (nylon; diameter 25 mm, pore size 0.45 μm, Merck®, Brazil), membranes were submerged in three solutions of acetonitrile:water (50:50, v/v) containing 14, 145 and 220 μg/mL of benzoyl peroxide and maintained at 32 ± 1 °C for 6 h. A control consisted of the same test solutions but with no membrane added, maintained under the same conditions of time and temperature [2,3]. Following the 6 h incubation the membranes were removed and the solutions assayed for the concentration of benzoyl peroxide remaining in solution. The recovery for each concentration was expressed relative to the control (dividing the concentration obtained for each solution containing the immersed membranes by the concentration obtained for the control solutions, subsequently multiplied by 100) [2,3]. The results are shown in **Table SI3**. All recoveries were within the range of 95.0 to 105.0%, demonstrating that there was no significant interaction between the benzoyl peroxide and the synthetic membrane chosen for the conduct of the IVRT [2,3].

Table SI3. Relative recovery of benzoyl peroxide from test solutions following incubation with the nylon membrane.

Test solution (μg/mL)	% Recovery
14 (LOQ)	99.3
145	98.5
220	99.0

In order to assess the stability of the benzoyl peroxide in the acetonitrile:water (50:50, v/v) receptor medium, three solutions containing benzoyl peroxide at concentrations of 14, 145 and 220 μg/mL were prepared as for the IVRT experiments. After preparation, an aliquot (5 μL) of each solution was immediately injected into the HPLC with the remaining solutions maintained at 32 ± 1 °C. This was repeated at defined times shown in **Table SI4**. The stability of the benzoyl peroxide in the acetonitrile:water (50:50, v/v) receptor medium solutions was evaluated by comparing the concentrations of benzoyl peroxide measured at each timepoint compared to the initial concentrations measured at timepoint zero [2,3].

Table SI4. Stability results for benzoyl peroxide concentrations over time in acetonitrile:water (50:50, v/v).

Test solution	Stability time evaluated	Recovery
14 µg/mL	12 hours	98.1%
	24 hours	96.9%
	36 hours	97.1%
145 µg/mL	12 hours	98.1%
	24 hours	97.4%
	36 hours	98.0%
220 µg/mL	12 hours	99.6%
	24 hours	98.9%
	36 hours	98.4%

As shown in **Table SI4**, all recoveries were within the range of 95.0 to 105.0%, demonstrating that the benzoyl peroxide, diluted in the receptor medium used for the IVRT (acetonitrile:water (50:50, v/v)) is stable up to 36 hours, maintained at 32 ± 1 °C.

Linearity, precision and reproducibility

Following the Food and Drug Administration (FDA) guideline "*In Vitro* Release Test Studies for Topical Drug Products Submitted in ANDAs" of 2022[5], it is possible to confirm linearity where cells have R^2 results greater than 0.97. In the IVRT experiments reported here, for all IVRT runs performed, R^2 was greater than 0.9. demonstrating that the release rate of benzoyl peroxide from the different evaluated matrices (reported in **Figure SI1**) follow the Higuchi model and present a linear relationship between the amount of benzoyl peroxide released and quantified in the receiving medium per unit area (mg/cm²) as a function of the square root of time in hours [2,3].

In order to evaluate the precision and reproducibility of the IVRT method, three experiments were undertaken using formulation A, containing 5% benzoyl peroxide, two performed on the same day (day 1) and one performed on day 2. The 18 resulting slopes (API release rates) were obtained from the linear regressions. Coefficients of variation (CV) for intra and inter runs were evaluated. Mean benzoyl peroxide release rate for each experiment was: 568.0 ± 10.1 µg/cm²/$h^{1/2}$ (day 1), 552.1 ± 10.4 µg/cm²/$h^{1/2}$ (day 1 repeat) and 591.4 ± 31.0 µg/cm²/$h^{1/2}$ (day 2) resulting in a CV of 2.0% for intra-run precision and a CV of 5.2% for inter-run precision within the acceptance criteria of ≤ 15%.

This provides evidence that the developed method presents satisfactory precision and reproducibility [2,3].

Sensitivity and selectivity

Sensitivity of the developed IVRT method was evaluated. The average release rate for benzoyl peroxide from two formulations containing 5% and 10% of benzoyl peroxide were compared (formulation A and formulation D), respectively (**Figure SI1**).

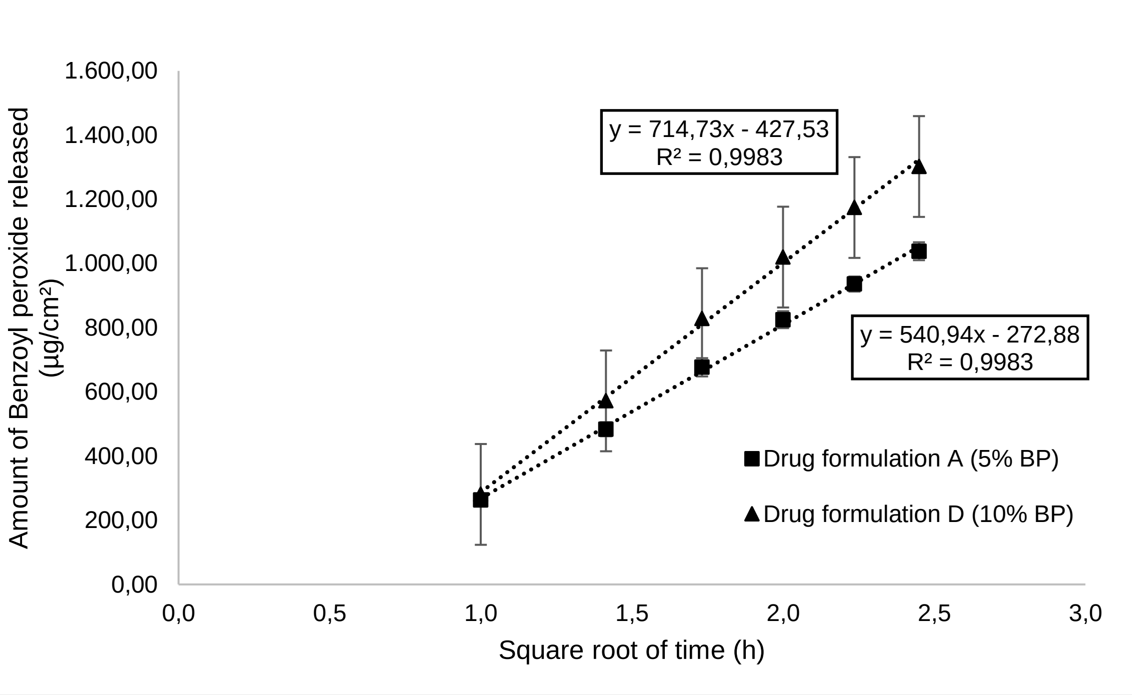

Figure SI1. Average release profile for drug formulations A and D, containing 5% and 10% of benzoyl peroxide, respectively. Data represents mean ± SD of 6 replicates, and the linear regression of mean.

An average release rate of 540.9 ± 9.6 µg/cm²/h$^{1/2}$ was obtained for drug product A (5% benzoyl peroxide) and 714.7 ± 25.9 µg/cm²/h$^{1/2}$ for drug product D (10% benzoyl peroxide), demonstrating that the developed method has adequate sensitivity to differentiate the average release rates from these two formulations [2,3]. In addition, the selectivity of the IVRT method was validated by use of a Mann-Whitney U test, described in the USP Pharmacopoeia[6], where the similarity of the tested drug products A and D we shown to be dissimilar (**Table SI5**). Formulations that present results within the acceptable range of 75-133.33% are considered similar[6].

Table SI5. Evaluation of the similarity of the tested formulations using the Mann-WhitneyU test.

Pairwise comparison	CI 90%		Sameness confirmed?
	Lower limit	Upper limit	
Drug product A vs Drug product D	129%	136.90%	No

The results presented in **Figure SI1** and **Table SI5** demonstrate the IVRT method developed has satisfactory sensitivity and selectivity [2,3].

Recovery

In order to characterize the depletion of the dose, the recovery was calculated according to **Equation 1**:

Equation 1:

$$\text{Recovery (\%)} = \frac{Amount\ of\ benzoyl\ peroxide\ in\ receptor\ solution\ at\ the\ last\ point\ (time{=}6\ h)}{Dose\ amount\ x\ product\ strenght}$$

The recoveries obtained for the IVRT experiments conducted for formulations A, B and C were 2.8 ± 0.1%, 1.6 ± 0.1%, 1.8 ± 0.1%, respectively. Therefore, it is possible to conclude that the developed IVRT method meets the premises established by the Higuchi model, since all recoveries obtained showed results ≤ 30% [2,3].

Supplementary References

1 ICH. ICH Q2(R2) Guideline on validation of analytical procedures. 2023; [cited 2024 Jun 16]. Available from: https://www.ema.europa.eu/en/documents/scientific-guideline/ich-q2r2-guideline-validation-analytical-procedures-step-5-revision-1_en.pdf

2 Tiffner, K. I.; Kanfer, I.; Augustin, T.; Raml, R.; Raney, S. G.; Sinner, F., "A comprehensive approach to qualify and validate the essencial parameters of an in vitro release test (IVRT) methdo for acyclovir cream, 5%.," *International Journal of Pharmaceutics,* 2018.

3 Purazi P, Rath S, Ramanah A, Kanfer I. Assessment of "sameness" and/or differences between marketed creams containing miconazole nitrate using a discriminatory in vitro release testing (IVRT) method. Sci Pharm. 2020 Mar;88(1). DOI: 10.3390/scipharm88010006.

4 EMA. Draft guideline on quality and equivalence of topical products. 2018.Available from: www.ema.europa.eu/contact.
5 FDA. In Vitro Permeation Test Studies for Topical Drug Products Submitted in ANDAs Guidance for Industry DRAFT GUIDANCE. Available from: https://www.fda.gov/drugs/guidance-compliance-regulatory-information/guidances-drugs.
6 United States Pharmacopeia - USP 2023, “Semisolid Drug Products - Performance Tests,” 2023.
